# Self-Presentation Concerns Among Injured Adolescent Athletes: A Qualitative Investigation

**DOI:** 10.3390/ijerph22111687

**Published:** 2025-11-07

**Authors:** Noémie Demers, Leslie Podlog, Lucie Forté, Alexis Ruffault, Marie-Lyne Nault, Jeffrey G. Caron

**Affiliations:** 1 School of Kinesiology and Physical Activity Sciences, Faculty of Medicine, University of Montreal, Montreal, QC H3T 1J4, Canada; leslie.podlog@umontreal.ca (L.P.); jeffrey.caron@umontreal.ca (J.G.C.); 2 Azrieli Research Centre CHU Sainte-Justine, Montreal, QC H3T 1C5, Canada; 3 Faculty of Sports Science and Human Movement, CreSco Toulouse University, 31062 Toulouse, France; lucie.forte@univ-tlse3.fr; 4 House of Performance, CREPS Toulouse, 31400 Toulouse, France; 5 Laboratory Sport, Expertise, and Performance (EA 7370), French Institute of Sport (INSEP), 75012 Paris, France; alexis.ruffault@insep.fr; 6 Department of Surgery, Faculty of Medicine, University of Montreal, Montreal, QC H3T 1J4, Canada; 7 Centre for Interdisciplinary Research in Rehabilitation of Greater Montreal, Montreal, QC H3S 2J4, Canada

**Keywords:** adolescent, sport injury, rehabilitation, return to sport, parents, coaches

## Abstract

Sport psychology research has shown that athletes might experience self-presentation concerns. However, fairly limited work has examined these specific concerns among athletes experiencing an injury, particularly among adolescent populations. Therefore, the purpose of this qualitative study was to explore the nature, precursors, and implications of injured adolescent athletes’ self-presentation concerns. Semi-structured interviews were conducted with female (*n* = 12) and male (*n* = 2) competitive adolescent athletes (*M*age = 15.1 years) who experienced a variety of serious injuries (e.g., ACL rupture, labrum tear) as a result of competing in various sports. Braun and Clark’s thematic analysis (2006) was used to develop themes pertaining to the nature, precursors, and implications of injury. Findings highlight a range of specific types of self-presentation concerns (e.g., concerns over “faking” an injury, lacking capability, disappointing others), the impact of the closeness of relationships with significant others, key implications (e.g., future sport apprehensions, negative emotions, motivational enhancements), and coping strategies. Results identify factors for targeted interventions aimed at managing self-presentation concerns among injured adolescents.

## 1. Introduction

Sport injury may have wide-ranging impacts on athletes’ physical, social and psychological functioning. Physical incapacitations such as immobility, reduced range of motion, tissue damage and swelling may be common [1]. Injured athletes also typically experience various social challenges including (but not limited to): pressures to return to sport from external sources—such as coaches, teammates and training partners—that can lead athletes to expedite their rehabilitation process and risk further injury [2,3]. Additionally, feelings of isolation and lack of social support from relevant others (e.g., coach, teammates, training partners) may hamper rehabilitation efforts [4,5]. For example, insufficient or inappropriate rehabilitation advice or guidance from coaches or sport medicine providers can reduce the chances of achieving optimal clinical outcomes (e.g., muscular strength, endurance, reductions in re-injury risk [6,7,8]).

In addition to the aforementioned social challenges, sport injury may induce a plethora of psychological difficulties. For many athletes, injury can impact their sense of self, leading to a decrease in self-esteem and confidence [9,10,11]. Injured athletes may also experience worries about regaining pre-injury capabilities, frustration over missed opportunities and concerns about falling behind peers [12]. Finally, emotional disturbances such as feelings of loss, denial, anger, depression, and fear of pain and re-injury are normal responses following injury [4,13,14,15,16,17].

While the various psychosocial challenges associated with sport injury have been examined among adult athletes, comparatively little empirical attention has been given to the adolescent sport injury experience. This relative lack of research is somewhat surprising, given that adolescence can be a tumultuous developmental phase, characterized by heightened emotionality, self-regulation issues, identity concerns, and difficulties associated with social skills acquisition [18,19]. Adolescence is also a development stage in which young individuals explore and reflect on their identity and how they hope to relate to the social world around them [20]. Compared to adults, adolescents place greater importance on peer relationships (e.g., acceptance and group identification) and frequently rely on peer feedback to set goals [20,21,22,23]. As a result, adolescents are especially sensitive to peer evaluations and are highly influenced by those around them. They often prioritize their public image and social acceptance, driven by a strong desire to make a positive impression and gain approval from their peers [21,22,24,25].

Given the aforementioned issues, it is not entirely surprising that initial research supports the suggestion that adolescent athletes may be particularly vulnerable to the adverse implications of injury relative to their older counterparts [18,19,26]. For instance, adolescent athletes have indicated experiencing more emotional disturbances, increased perceptions of post-surgical pain and state anxiety, a higher reliance on social support after injury, and poorer rehabilitation adherence compared to adult athletes [27,28,29,30]. Further, young athletes who sustain injuries—in particular those with a strong athletic identity—may experience elevated depression [6,31,32] and psychological distress, including symptoms of avoidance, intrusion, and hyperarousal [33,34]. Consistent with findings among adult athletes, concerns about pain, re-injury, and a loss of physical fitness along with worries about falling behind others, missing out on sport/social opportunities and underperforming, have been found in injured adolescent athletes [16,17].

One psychological challenge that may be particularly germane among injured adolescents, but which has received scant empirical attention, relates to self-presentation concerns. Self-presentation, also referred to as impression management, refers to concerns about how one is perceived by relevant others and associated attempts to influence such perceptions [35,36]. Self-presentation is a natural and essential aspect of human interpersonal behavior [35]. While most people are aware that they are being evaluated by others, not everyone is equally concerned with such evaluations.

Research by Podlog, Dimmock and Miller [37] indicated that injured athletes often experienced self-presentation concerns such as appearing unfit or unskilled, particularly after a prolonged absence from sport, and face uncertainty about meeting others’ performance expectations. These concerns were heightened in athletes who were more aware of the attention from coaches, teammates, and fans regarding their return to sport [38]. Additionally, athletes may engage in strategic behaviors to present themselves differently to others to compensate for their injury and thus diminish their self-presentation concerns. Bailey et al. [39] supported findings by Podlog and Eklund [38] revealing that individuals with spinal cord injuries, particularly women, were preoccupied with maintaining an image of physical attractiveness and used strategies such as makeup, jewelry, and clothing to enhance their appearance. They also engaged in tactics to appear confident and independent, avoiding the perception of being lazy or inactive [39]. Further, findings from Driediger et al. [40] indicated that self-presentation concerns were prominent among both healthy women presented with hypothetical rehabilitation scenarios and injured women at the onset of rehabilitation—regardless of age, fitness level, or social physique anxiety. While the aforementioned findings provide an initial understanding of self-presentation concerns in rehabilitation, results suggest that these concerns can negatively impact the rehabilitation process.

Of the limited research on self-presentation concerns among injured adolescents, a two-study publication by Podlog et al. [41] suggested that injured high school (male = 61, female = 57) and collegiate athletes (male = 62, female = 43) with high self-presentation concerns and strong athletic identity were more likely to engage in risky rehabilitation behaviors. Behaviors included over-adherence (rehabilitation efforts exceeding practitioner recommendations) and prematurely returning to sport if they believed it would earn social approval. Self-presentation concerns, such as fear of appearing athletically untalented, physical appearance, and fatigued/lacking in energy, were also linked to ignoring practitioner recommendations. A key finding was that adolescent athletes were more concerned about their athletic appearance compared to collegiate athletes, suggesting their particular vulnerability to self-presentation pressures.

Given the limited research on self-presentation concerns, particularly among adolescents, and the predominance of quantitative work, there remains a lack of qualitative insight into the types of self-presentation concerns relevant to adolescent athletes. Additionally, most prior research has been retrospective, requiring participants to reflect on past experiences, thereby introducing potential retrospective recall bias. To address these gaps, qualitative research involving currently injured adolescent athletes is needed to capture their lived experiences. In light of the aforementioned limitations in previous research, the aim of the present study was to examine the nature of adolescent athletes’ self-presentation concerns, the intra- or interpersonal factors which precipitate such concerns and the potential implications of self-presentation concerns for adolescent athletes’ rehabilitation and return to sport. Considering the qualitative nature of our investigation, we did not advance directional hypotheses. That said, we anticipated that a range of self-presentation concerns would be evident among adolescents and that such preoccupations might hold important implications for adolescents’ rehabilitation and return to sport.

## 2. Materials and Methods

### 2.1. Study Design

Underlying assumptions about reality (ontology) and knowledge (epistemology) represent a researcher’s (or research team’s) philosophical positioning, a set of beliefs that underpin the design and execution of a qualitative study [42]. This research was approached from a critical realist paradigm, adhering to a critical realist ontology (i.e., presence of multiple realities grounded in participants’ experiences) and subjective/transactional epistemology (i.e., knowledge is created through interactions with participants and interpretations of the transcript data are influenced by researchers’ previous experiences) [43,44]. Therefore, knowledge generated from this study is co-constructed by participants and researchers.

### 2.2. Procedure

After obtaining University ethics approval (2024-5666) and that of the Sainte-Justine University Hospital Center (2025-7922), sport science professors and contacts at various training centers and sport organizations across Quebec and France were contacted and invited to share a description of the nature and aims of the study with potential participants. Eligible participants were either referred by professors and contacts or directly contacted by the primary investigator, who then provided participants with an informed consent form and demographic questionnaire via email and online link. Participants were offered various time slots (accounting for time zone differences) before a mutually convenient time and date was agreed upon for each interview.

### 2.3. Participants

To be eligible for study involvement, participants were required to meet several criteria: 1. Adolescents aged between 12 and 17 years old. 2. French speaking. 3. A competitive youth athlete—that is competing at a regional, national, and/or international level. 4. Have sustained a severe sport injury requiring a minimum 4-week absence from sport-specific training or competition [45,46]. 5. Currently undergoing some form of rehabilitation. 6. Express an interest in returning to sport participation following injury rehabilitation. These inclusion criteria were chosen to ensure that participants could provide in-depth and rich detail about their self-presentation concerns in injury rehabilitation and return to sport experiences. Participants interested in the study were sent an information and consent form via email, which they reviewed and signed prior to participating in the interview. As minors were included in this study, parents’ review and signature of the informed consent sheet was mandatory for participants’ participation. Consent was considered obtained upon returning the signed form by both parents and participants. Participants were also asked to complete an online demographic questionnaire (approximately 5–10 min to complete) before participating in the interview. Our final sample included fourteen male and female adolescent athletes (*n* = 2 males; *n* = 12 females; *M*age = 15.1) from Canada (*n* = 12) and France (*n* = 2) competing at regional (*n* = 4), national (*n* = 7), and international levels (*n* = 3) who volunteered to participate in the study. We continued recruiting participants until we reached data saturation, that is, the point at which no new themes emerged [47]. Athletes were competing in team sports (basketball: *n* = 2, cycling: *n* = 1, ice hockey: *n* = 1, soccer: *n* = 4, softball: *n* = 1) and individual sports (acrobatic skiing: *n* = 1, diving: *n* = 1, gymnastics: *n* = 1, judo: *n* = 2). At the time of interviews, participants’ absence from sport training and/or competition represented approximately 25 weeks (*M* = 24.41 weeks, range = 4 to 67 weeks). Athletes experienced a variety of injury types including (but not limited to): anterior cruciate ligament tear, labrum tear, stress fracture and elbow sprain. Participants were assigned a pseudonym and any in-quote information (e.g., name of sport team, team organizations, locations) that could compromise their anonymity or confidentiality was modified. Participants’ demographics are presented in Table 1.

### 2.4. Qualitative Data Collection

Consistent with our critical realist approach, we gathered data by conducting semi-structured interviews [43]. All interviews were conducted by the first author and took place virtually via a videoconferencing software (i.e., Microsoft Teams). Interviews were audio-recorded for transcription purposes. As interviews were conducted in French, all French transcripts were translated into English prior to data analysis.

**Figure 1:**
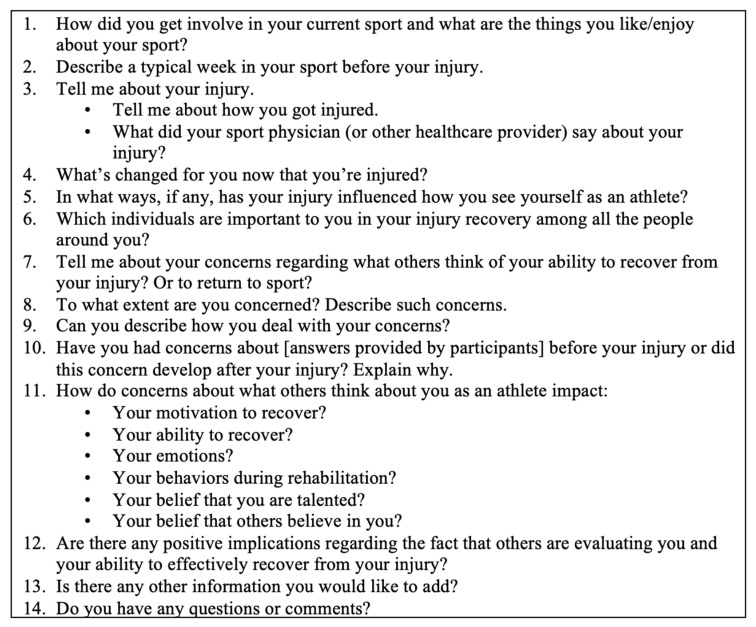
Interview Guide.

A semi-structured interview guide was developed in order to address key research aims [48]. Following rapport-building questions regarding participants’ overall sport involvement (e.g., “How did you get involved in your current sport and what do you love about it?”), participants were asked about the nature of self-presentation concerns (e.g., “Tell me about your concerns about what others think about your ability to recover from your injury?”), the precipitating factors of such concerns (e.g., “Why do you have these concerns?”), and potential implications for their rehabilitation and return to sport (e.g., “How might concerns about what others think of you as an athlete impact your motivation to recover?”). Interview questions were developed based on previous research examining injured athletes’ self-presentation concerns, the aims of the current work, and theoretical tenets associated with the construct of self-presentation [35,40,49]. Participants were also encouraged to discuss any experiences or raise any relevant issues not addressed in the interview guide. Although the interview guide provided a structured set of questions, the order of questioning varied across participants according to their responses and the flow of conversation. Thus, the interview guide provided consistency while allowing for flexibility and expansion through probes to support more detailed responses from participants [48]. A full list of interview questions appears in Figure 1.

### 2.5. Data Analysis

On average, interviews lasted 38 min (range = 20 to 55 min) and resulted in a total of 123 pages of single-spaced transcription. Transcripts were stored and organized in NVivo 15 (a qualitative data analysis software). In line with our philosophical positioning, a 6-step thematic analysis was undertaken to interpret subjective experiences of participants in relation to the research questions [50,51]. For the first step, the first author familiarized herself with the data by listening to recordings, transcribing interviews and reading through the transcripts. Additionally, during the first step, the first author annotated the transcripts, highlighting consistent information throughout all transcripts. For instance, the first author noted in the margin: “presence of relevant others in athlete’s injury experience (i.e., parents, teammates, coaches)” and “closeness of coach-athlete relationship seem to accentuate participants’ concerns”. Second, all transcripts were coded inductively and deductively by assigning labels relevant to answering our research questions. For example, the following quote: “Now, we have a new coach who has just arrived […] it stressed me out a little for the practice afterwards because I was like… She’ll think I’m not good” was coded “coach-athlete relationships (i.e., new coach) accentuated self-presentation concerns”. The third step consisted of creating themes by grouping codes into “sub-themes” and then “higher order themes” using a back-and-forth iterative process. For instance, the sub-theme, “impact of the closeness of relationship with significant others on self-presentation concerns” was categorized under the predetermined higher order theme “factors influencing self-presentation concerns.” Fourth, each theme was reviewed, with the intent of ensuring that all created themes represented an accurate representation of participants’ statements and experiences. Fifth, to clarify and operationalize each sub and higher-order theme, definitions of each sub-theme were created. For example, the definition of the sub-theme previously mentioned was: “the degree of familiarity in relationships with others, influenced injured adolescent athletes’ self-presentation concerns.” In the sixth and final stage, each theme was contextualized and elaborated on by including participant quotes.

### 2.6. Methodological Rigor

To enhance the rigor of our analytic process, the use of two procedures was adopted. First, the lead author critically engaged in reflexive memos throughout the analysis process, reflecting on initial interpretations of the data and highlighting comparisons between participants’ experiences. For example, the following memo illustrated the first authors’ reflections: “some athletes worried about being seen as “faking” their injury, but the reasons behind this concern varied. For example, one participant felt this perception came from others thinking they were trying to avoid training, while others believed it was tied to not having a long-standing relationship with their coach.”

Second, the use of “critical friends” was implemented to challenge ideas, consider different interpretations and encourage reflection among members of the research team [52]. For instance, through the process of data analysis, the first author presented coded data and emerging themes to the second author and other lab colleagues. During meetings, critical feedback, reflexive insights, and probing questions were offered to challenge and refine the first author’s interpretations (i.e., construction of knowledge). The primary aim of the “critical friends” process was to encourage reflection, explore different perspectives, and foster discussion to reach a shared understanding and agreement of theme selection [52]. Importantly, these meetings also provided the opportunity to explore other valid ways of making sense of the data and to defend previous interpretations. Additionally, all co-authors contributed feedback and reflections on the coherence and logical consistency of the developed themes.

## 3. Results

Three main themes consistent with our a priori research questions regarding the nature, precursors, and implications of self-presentation concerns were developed. In addition to these three themes, a fourth theme pertaining to the strategies athletes used to manage self-presentation concerns emerged. Each theme and relevant sub-themes are described below.

### 3.1. Nature of Self-Presentation Concerns

Participants reported several specific self-presentational concerns including: (1) concerns that relevant others believed they were “faking” an injury or using it as an excuse; (2) concerns over lacking capability in the eyes of others; and finally, (3) concerns over disappointing or letting relevant others down.

#### 3.1.1. Concern over Being Perceived as “Faking” an Injury or Using It as an Excuse

A key preoccupation for athletes was that individuals whose opinions they valued would believe they were “faking” their injury. Epitomizing this self-presentation worry was the comments of Margot, who stated: “Well, sometimes, I’m afraid that [my parents] think I’m lying or that I’m acting. It’s true that I’ve been injured for so long.” Interestingly, the length of the injury seemed to play a role in fostering this concern. Margot mentioned having consulted various specialists over several months, all of whom were unable to identify her injury, and with little improvement in her condition, her parents started doubting the presence of her injury. Reflecting on her brother’s perception of her during her rehabilitation period, Margot emphasized the notion of time: “It’s true that I’ve been injured for so long that many people often think it’s cinema…” In support of this idea, Valerie remarked: “Not necessarily my parents, but let’s say my head coach in soccer, there are other people who don’t know me as well. Now, since November, I haven’t been there. I’m afraid people will think that… Not that I’m faking it, but like a little.” For Valerie, both the duration of the injury and the closeness of her relationships seemed to be related to her injury faking concern. While her extended absence seemed to raise doubts, Valerie suggested that she could explain her situation to people who knew her well—such as close friends—which alleviated her concerns. In contrast, among those less familiar with her, Valerie reported that she lacked the opportunity to address her situation, which heightened her concern of being perceived as “faking” her injury.

Some participants expressed a concern over being perceived as using their injury as an excuse to avoid training in front of others. For Olivia, this apprehension was reinforced by her teammates’ comments that she was “faking” her injury to avoid physically demanding sessions. She suggested that: “Sometimes, [my teammates] don’t do it on purpose. They know that it pisses me off… Then, they told me: ‘We know that you’re faking just so you don’t do the laps of the pitch because it’s demanding, it’s hot.’” Likewise, Emily expressed worry about being misjudged by a new coach—a figure unfamiliar with her previous commitment to training. This lack of an established relationship heightened her worry of being perceived as less dedicated to her training or as avoiding hard work due to her injury compared to teammates. Emily shared: “I’m a little worried that she thinks I’m a coward, that I don’t want to train as much as the others.”

#### 3.1.2. Concern over Lacking Capability

For some participants, a key source of unease was related to perceptions of competence, specifically about being seen as less capable due to their injury. This concern pertained to athletes’ diminished sense of ability such as being seen as weak, less capable, or not performing at their previous level. A frequent misgiving related to being perceived as “weak” or “a quitter”. To illustrate this concern, Margot shared her apprehension about how others might have interpreted her decision to step away from her sport because of her injury: “Yes, that’s right, I’m afraid that people will think that I was the one who decided to give up, because it’s too hard…” Emma echoed similar concerns, particularly about others seeing her as someone who would regress or whose season was over due to injury: “Yes, I don’t really like it when people think that of me, that they think I’m a bit of the weakest link, let’s say.” She continued: “I don’t like it when people have the image of me as someone who always needs help from others, who is always injured…[always] on the edge, watching others. Likewise, Marie reflected on the stigma athletes may face upon returning to competition: “When you see someone coming back from an injury, they can be considered weak at first. So, for me, it was really more these worries like that.”

Additionally, some participants highlighted discontent over being perceived as “useless” or “unreliable” during and after their injury. Emma shared her discomfort with needing assistance from others, particularly in everyday situations like having teammates help carry her bag at school: I don’t like [needing help from others], it makes me feel useless.” Margot reflected on similar concerns, particularly in the context of her coaches’ decisions regarding her future in the sport. She worried about no longer being able to fulfill her previous leadership role on the team and about being viewed as dispensable: “And so, I’m afraid that now they will say: ‘Oh finally, she’s useless now, we can’t even count on her to be in front.’” Margot also suggested that her prolonged injury might affect future selection decision, suggesting that she might now be seen as unreliable: “In fact, I’m afraid, for example for the next selections, that they will say to themselves: ‘We can’t count on her, because every time she gets injured and we’re not going to try to take her back.’”

As athletes turned their attention towards their return to sport following rehabilitation, some worried that others would question their ability to perform at pre-injury levels. The extent to which athletes worried about teammates versus coaches or other evaluations of post-injury performance differed from one participant to the next, depending on who they perceived as more influential. For example, Emma, who competed in an individual sport was primarily concerned with her coach’s perception of her post-injury capabilities: “I’m afraid he thinks I’m not going to go back to the way I was before, that I’ve regressed.”

However, for most participants, concerns centered around how teammates would evaluate their level of performance upon return. According to Marie:

It’s not necessarily that [my teammates] think they’re better than me, but the fact that I’m coming back to the game, in addition, after a long period of not playing, they may think that maybe I’m not at the level, that they can easily pass me or that I’m not the player I was before.

Similarly, Benjamin, who competed at a high level, highlighted the pressure to meet team standards: “I don’t know, because I’m playing at a high level. The standards are high. I don’t want [my teammates] to think [the] worse of me, like I’m not at their level or anything like that.” He further reflected on how his teammates might perceive his future performance: “…that maybe they’ll think when I get back on the pitch: ‘Oh, it’s going to be easy to play against him, because it’s been a long time since he’s played.’” Benjamin’s comments suggested a deep concern about regaining performance levels that matched the expectations of the competitive environment. Eve also shared similar concerns, even while acknowledging that negative comments would be out of character for her teammates:

I don’t think that’s their style, but sometimes to say a little comment behind my back: ‘Will she get back to the level?’ It’s more of a pressure to me. I start to think that they could say that about me. It’s more like that, but it’s not their style, but still, it’s more… My friends, that’s it… Could they think I’ve gotten worse? When I come back to the game, they’ll make fewer passes to me or things like that. I’m not really sure.

Interestingly, Eve not only worried about how she might be perceived, but also about possible behavioral changes from her teammates—such as being passed the ball less—suggesting a potentially deeper concern about social reintegration into the team.

For some participants, it was not particularly clear whom they were referring to when expressing concerns about others’ perceptions, indicative of the fact that these concerns extended to a broad range of individuals in their sporting environment. For instance, Eve stated:

I have the impression that when I come back to the game, people will be very fixed on: ‘What is she doing? Will she be able to do what she did before?’. Especially things like that. ‘Will she come back to her best form? Will she be able to make as many goals or as many saves as she did before?’ It’s more like that.

In the case of Evelyn, she expressed that others had high expectations regarding her performance upon returning to sport. As she explained: “Of course, I was starting from the top. I had won quite a bit all year. I thought: ‘People know I’m supposed to be good.’” These expectations seemed to contribute to her concern about the potential negative evaluations of her performance after a prolonged absence. Evelyn added: “Of course, there may have been some who were thinking: ‘She’s been held back for a long time, maybe [her performance] is going to be [not as good as expected].’” Similarly, Valerie expressed a concern about being judged by others upon her return: “Yes, I’m afraid people will say: ‘Come on, she’s lost some.’” Participants’ comments suggested a general concern about being negatively evaluated by a wider audience–not just coaches and teammates, but potentially peers, parents, spectators, or other members of the sporting community–regarding their ability to regain prior performance levels.

#### 3.1.3. Concern over Disappointing or Letting Others Down

A common source of distress among athletes was related to worries about failing to meet the expectations and hopes of others, thereby disappointing those who were significant to them. This worry was evident both during rehabilitation and when participants considered their future sport performance. During rehabilitation, athletes experienced heightened apprehension about how their efforts and progress were perceived by others. For Margot, this concern involved being seen as lacking motivation or not putting in enough effort. For instance, Margot expressed being worried about disappointing her parents, who seemed to be expecting her to remain committed to her sport and actively working toward recovery. Margot detailed:

Yes, but also, I’m afraid that [my parents] will think I’m not interested in cycling anymore. I don’t know how to explain, because there were times when I clearly gave up. There was a session to do, but I didn’t do it, because I was in too much pain, but I don’t want them to think it’s because I don’t have a mental strength or that I don’t care about cycling anymore.

For some participants, this concern extended to healthcare providers. Charlotte described the pressure to demonstrate progress and meet her physiotherapist’s expectations over her recovery: “Of course, I feel worried, because I don’t want them to tell me: ‘Actually, [your recovery] is not as good’, but I always try to get to a certain level so that they are happy with the progress.” Valerie also shared a similar experience, noting that her previous recoveries were quick and linked to her effort levels, which added pressure to her current rehabilitation which was taking more time than previous injuries due to her lack of adherence to the prescribed exercises. She mentioned:

Well, let’s say, I’ve had other injuries before. I was able to really recover quickly. According to [my physiotherapist], I was able to because I did my exercises a lot. She pressures me to recover because [my current injury] is healing more slowly. Now, I’m afraid that she will think that I don’t care anymore, since I haven’t been doing [my exercises].

When envisioning their return to competition, several participants expressed some concerns about not meeting the performance expectations of others and, as a result, potentially disappointing relevant others. For Anna, worries of not living up to others’ expectations of her return to competition were particularly directed toward her parents. When asked about her parents’ expectations, it seemed difficult for her to articulate the emotional weight of their expectations and she appeared challenged when asked directly about it. To support her in exploring this concern further, the first author offered a reflective prompt: “…from what I understand is really that yes, maybe you can present yourself differently, because you didn’t want to disappoint them, something like that?” To which Anna answered:

Yes, a little. I knew I wasn’t going to disappoint them. I was hoping I wasn’t going to disappoint them, but I felt a little like… How can I put it? I felt that they expected a lot for my return.” Anna went on to explain: “I think they really believe in me. Maybe sometimes I had the impression that they thought they saw more than I did. I don’t know how to put it. We were talking about my first match that was coming up. I had the impression that they thought I was going to play better than I thought I was going to play, I don’t know how to put it… That stressed me out a little, because if I don’t play the way they think I should… It’s not like there will be any consequences, but I didn’t want to disappoint them, but in the end, it was my first match.

Along these lines, Charlotte shared: “I think I just want to… Make sure I don’t disappoint, because I was already at a good level, I just want to get back to the good level I was at. But I think that’s really just what might worry me… [Disappointing] my family, I guess.” Even in the absence of explicit pressure or potential retaliation, participants’ comments illustrated the complexity of navigating others’ expectations about their return to competition.

For some participants, the concern about disappointing relevant others was directed to their coaches. Benjamin shared an apprehension about being seen by new coaches for the first time post-injury, as they had never witnessed his performance prior to his time away from sport. At the time of injury, he had just joined a new team, making the situation more difficult: “I’m afraid they’ll see the first time I’ve practiced, because it’s been a long time since I’ve touched the ball, I’m not what they expected, what they expected of me.” Evelyn also reflected on her coach’s potential disappointment over not meeting prior standards of performance: “I was a little sad because I was thinking: ‘He’s going to be a little disappointed, because I can’t compete like I used to.’” In the case of Eve, she also discussed concerns about both her coach and her family. She emphasized a desire to remain a role model within her sport and to live up to the support she had received:

Of course, I’ve had a lot of… not favours, but for sure I’m really appreciated, I think, by my coaches. Of course, I wouldn’t want to… I’m not going to want to disappoint them when I come back, as well as my family, because they’ve put so much effort into me the last few years to follow me through my passion, and all that. I don’t want to disappoint them, because I’m not as good as I was before. I think I’d put it that way. I don’t want to… I’d like to remain a model of what I was and what I am in my sport.

When athletes reflected on the possibility that their rehabilitation might not go as hoped and that returning to sport could become uncertain or even impossible due to injury, some athletes expressed a deep concern about disappointing or letting down their families. This concern was particularly tied to parents, especially given the time, energy, and sacrifices their families had made to support them in their sport career. The prospect of being unable to continue their athletic journey or achieve long-held dreams led to worry of letting their families down. Emily shared:

I still feel bad, because they put up with everything. Before, it was… Thanks to my mother that I was able to leave [my hometown]. I say to myself: ‘Ah, it would be sad if all this led to me not achieving my goals’. […] but of course yes, it worries me a little in that sense. Just the fact that they all put up with me, and let’s say it [my career] all stops, that would be a bit sad really.

In a similar vein, Benjamin said: “I think [my parents] want to see me do well and everything. That’s why I don’t want to let them down.” These reflections suggested that the concern of disappointing relevant others extended beyond individual performance and seemed tied to a potential premature end to one’s athletic journey.

### 3.2. Factors Influencing Self-Presentation Concerns

During the course of interviews, participants were asked about factors that they believed influenced the extent to which they experienced self-presentation concerns. A key consideration that was repeatedly highlighted pertained to the closeness of the relationships that athletes shared with relevant others, a theme expanded on in greater detail below.

#### Impact of the Closeness of the Relationship with Significant Others on Self-Presentation Concerns

Many participants discussed the connection between the extent to which they “knew” relevant others in their sporting environment and the degree to which they experienced self-presentation concerns. For some participants, self-presentation concerns were greater in relation to individuals they were less close to. It appeared that unlike close relationships, where open communication was possible, distant or semi-acquaintance relationships often left little room for athletes to explain their situation. Eve expounded on this idea, describing how others’ assumptions, especially from classmates, negatively impacted her:

I’ve always been very affected by what people might think, even before my injury. I would say that, precisely in relation to that, it’s more the people I don’t know very well that I’m afraid of what they might say… Like people not so close to me, my classmates or people like that. I’m not too close to them. What could they say about [my situation]? What is she doing? What’s her injury? What if she is never going to be able to play soccer again or things like that?”

Eve further suggested that classmates’ lack of familiarity with her made her feel she had more to prove: “Maybe because I have the impression that they know me less. It’s more worrying to have to prove to them that I’m going to be resilient, that I’m going to be able to develop even after my injury.” Marie too shared that the opinions of those who were not close to her, and who lacked insight into her daily struggles during injury, had an impact on her:

I would say that it still had an impact… I think it’s mostly people who weren’t… Who didn’t know, who weren’t really close to me and who didn’t see my reality, what I was going through every day, how it was mentally or not. I really thought more about these people.

However, for others, familiarity seemed to act as a protecting factor, reducing self-presentation concerns. Knowing other individuals well provided reassurance that such persons would not negatively judge or evaluate them. It appeared that with individuals close to them, some athletes felt better able to explain their situation and share their injury experience. Being able to communicate openly about what they were going through appeared to diminish self-presentation concerns. As Valerie remarked, “Yes, that’s right, because [people close to me], when they come to see me, then I explain to them, then they understand. But let’s say, sometimes, people who don’t know me, they don’t know that it’s not really like me to fake something like that.” During her interview, Valerie clarified the difference between teammates she felt close to and those she did not when probed by the first author: “Earlier, you said that you had fewer worries about the teammates you have at your age, at your level. Why do you think it’s different, that you have concerns about older girls, but not necessarily with girls your age?” to which Valerie responded: “Because I’m really closer to girls my age. Girls my age… How could I put it? Yes, I’m closer to girls my age. I’m not really afraid that they’ll think I’m really faking, because they really know me better.” In a similar fashion, Emily shared that having a long-standing relationships with her teammates helped alleviate her concerns: “I don’t have the impression that they think negative things, because I’ve been with them [for] a long time, I’ve been training with these girls [for] a long time, they know me, they know, that’s why I don’t have any concerns.” Eve echoed this sentiment, emphasizing how open communication with those she was close to helped her feel understood and supported: “I have the impression that since I talk more with the people close to me, my family, my friends, my coaches, than them, they really understand my way of thinking, and then my situation that is coming. They don’t make little “announcements” or rumors about me…”

On the contrary, some participants expressed greater concern about people they felt closer to as it seemed to accentuate their concerns–possibly because they placed greater value on those individuals’ opinions. For example, Evelyn shared that her self-presentation concerns were especially tied to the people in her close circle: “Probably the parents of other athletes, athletes, especially those in my close circle. I don’t think it would go as far as people like further [outside my close circle], but in my close circle.” She even noted that the parents of other athletes might feel “satisfied” if her return to competition was unsuccessful, making their perception of her more worrisome. Charlotte also mentioned being more affected by the opinions of those she was close to:

No, I think my concerns are more about those that I am close to than those that I am less close to. I’ve always been like that. It’s as if those I’m less close to… It’s like when you walk outside and you [come across someone you] don’t know very well, you tend to pay less attention to them and more to the person you’re walking with.

Interestingly however, Charlotte seemed to contradict the above statement at a later point during the interview, suggesting heightened concerns about the perceptions of her new coach, with whom she had not yet developed a close relationship: “Basically, I just changed coaches, which means… [I’m] less close to them, it’s just that… I don’t know them well enough, I’d say. We have just started the year, and because it’s new, you don’t know them as well…” Charlotte also wondered how her new coaches might perceive her future performance, given that they were not yet familiar with her capabilities: “Will I be strong enough to come back to the way I played before, and will they like it?”

The duration of the coach–athlete relationship appeared to be particularly important in determining the extent to which athletes ‘held reservations about coaches’ opinions of their sporting abilities. For some participants, not having a long-standing or close relationship with their coach contributed to heightened self-presentation concerns. For example, Emily described feeling worried about how her new coach, who had only seen her during her injury, might perceive her:

There’s a coach who arrived not long ago. Then she, as soon as she arrives, she sees that I’m injured. I don’t do training like other people, so she, honestly, yes, it’s true, I’m a little worried that she thinks I’m a coward, that I don’t want to train as much as the others. But that’s just because she’s new.

Emily further explained that these concerns would likely fade if the coach got to know her better. In contrast, she felt more confident in the perceptions held by a coach she had worked with for an extended period. Emily stated: “Well, that coach I told you about, him in [city]. I don’t think his perception of me has changed that much, because he’s been seeing me every day, many hours for 2 years. He knows that I want to give it my all…” Anna echoed a similar concern in the context of having a new coach: “Now, we have a new coach who has just arrived […] it stressed me out a little for the practice afterwards because I was like… She’ll think I’m not good. That’s her first impression of me.”

The importance attributed to coach evaluations of one’s abilities appeared to stem largely from the power and influence coaches held over athletes’ sporting futures. Indicative of this sentiment, Margot explained:

Well, […] especially in relation to, for example, the selectors for the Championships. Uh, I was very scared, and I was… Finally, I told myself that they must be thinking that in fact they had selected the wrong person and that if they had selected another girl, it would have worked or whatever. So, I think so. Their opinion is more important than those around me.

### 3.3. Implications of Self-Presentation Concerns for Athletes’ Rehabilitation and Return to Sport

Participants indicated that many of the previously described self-presentation concerns held a number of salient implications for them, namely concerns regarding their future sport participation and the experience of negative emotions. Conversely, it was also suggested that self-presentation concerns could positively impact athletes’ motivation to recover.

#### 3.3.1. Self-Presentation Concerns Leading to Apprehensions About Future Sport Participation

Participants relayed worries that negative coach judgments regarding their potential loss of ability could negatively influence key decisions such as their playing time and their potential role on the team when returning to sport. Such concerns were primarily directed toward coaches, rather than other relevant figures, given that coaches were believed to have the greatest authority over decisions about athletes’ future sport participation. As Benjamin explained: “The coach is more the one who makes the decisions, [for example] who plays and all that. That’s why I think he’s the most important.” Similarly, Charlotte noted: “Because they’re the ones who will decide my playing time. They are the ones who will decide if I play or not.” Anna clearly illustrated how concerns about her coach’s perception could affect her return to sport: “She’ll think I’m not good. That’s her first impression of me. After that, this summer, I hope she’ll put me on the field.” When the first author asked for further clarification, Anna added:

If she thinks I’m not as good, she’ll put me in the game less, for sure. The better you are, the more playing time you get. That’s right. I want her to have a good impression of me. That way, I’ll get more playing time. I’m not going to play soccer to be on the bench.

Some participants expressed worries about their role within the team upon returning to sport. For example, Eve acknowledged the competitive nature of soccer at her level and her fear about the decisions coaches might make regarding her place on the team: “Of course, soccer is so much a competitive sport that what scares me is what choice they’re going to make for me, and then for their team when I come back.” Similarly, Margot voiced concerns related to her injury history and how it might influence her coaches’ selection decisions: “I’m afraid… For example, for the next selections, that they will say to themselves: ‘We can’t count on her, because every time she gets injured and we’re not going to try to take her back.’” Margot comments epitomized athlete concerns about being perceived as unreliable and, as a result, being excluded from future team selections or losing their former role.

#### 3.3.2. Self-Presentation Concerns Leading to Negative Emotions

Participants noted that the self-presentation concerns described in sub-theme 1 often gave rise to negative emotions such as anger, fear, sadness and anxiety. As Eve shared: “I was a little sad because I was thinking: ‘He’s going to be a little disappointed, because I can’t compete like I used to.’” In some instances, self-presentation concerns evoked mixed or overlapping emotions in participants, for example, a combination of anger and sadness. Olivia stated: “I was sad, and I was a little angry at them for saying that, because I really wasn’t faking [my injury]”, while Eve mentioned: “I was more a little angry, borderline. I would say, yes, sadder, for sure, because I don’t like it when people think things like that about me, but angry, because they don’t know what I’m going through.” Athletes also described how self-presentation concerns led to subsequent stress. Benjamin described feeling significant stress when reflecting on his concern that his teammates might view him differently or see him as not being on their level. He stated: “When I think about [my teammates seeing me as someone different] too much, it just stresses me out.” For Charlotte, negative evaluations from others contributed to anxieties that intensified self-doubts about her abilities: “Because I think the concerns came with the stress of: ‘Am I going to become the way I was? Will I go back to the level I was?’” The experience of injury—and the resulting self-presentation concerns—appeared to trigger social anxiety in Eve. She revealed a personal history with anxiety, the latter of which demonstrated a complex relationship between her injury-specific, self-presentation concerns and pre-existing trait anxiety:

Of course, my anxiety didn’t help all those concerns. Really, on the contrary, I don’t think that… Before [my injury], I had a lot [of anxiety], but more in relation to the social aspect. In terms of my talent, I was always anxious about it. Making friends, and then all that, it wasn’t always easy for me. Before [my injury], I thought it was more about that. I was like able to fix everything, all this stress, this social anxiety. I was able to fix that, but now it seems like it’s coming back. It seems like it’s coming out a little bit in me since I had my injury. Of course, these concerns don’t help, and vice versa, anxiety doesn’t help concerns and then concerns don’t help remove anxiety either.

#### 3.3.3. Self-Presentation Concerns as a Source of Motivation

In addition to the negative implications of self-presentation concerns, participants also reported a number of positive motivational implications of such worries. Injured adolescents expressed a desire to prove others wrong, to show others that their negative evaluation of them was ill-founded and to work harder during their rehabilitation. For instance, when reflecting on his teammates’ perception of his inability to come back from his injury, Liam shared: “Well, I almost want to say, it affects me, but positively, because I just want to give more of myself to prove to them that they weren’t right, and that I was sure I was going to come back.” Similarly, Benjamin mentioned:

I think that when I think about other people thinking the worse of me, like I’m not at their level or anything like that, it’s a source of motivation, because it pushes me to prove them wrong, how can I put it? […] That is to say, to prove them wrong. I want to improve, as much as possible when I think about it.

Emma also commented: “Yes, I think that suddenly, it will make me want to prove them wrong, that despite the injury, we can come back much stronger…” In addition, Emma conveyed an optimistic vision of coming back from an injury: “…and that it’s not the end and it’s the beginning of a new path, let’s say, after we get back on our feet.”

For some participants, the desire to prove others wrong worked in combination with a heightened drive to work harder during rehabilitation. Liam’s comments nicely encapsulated a simultaneous interest in defying others’ expectations and working harder:

I would say that it gives me more motivation. It gives me more motivation… I know it’s kind of the opposite effect, but I’m the kind of person who, the more mean you are, the more bad things you’re going to do [to me] … I want to prove you wrong, but in a more positive way. I would say that it just pushed me to work harder.

Eve reflected on how external judgments served as a motivating force rather than a deterrent in her recovery: “I think on the contrary, the fact that people can say a lot of things… I find it’s a source of motivation to say: ‘It [what people say] is not necessarily true’. On the contrary, I can continue to work hard, and then I’ll get to the same goal I set for myself.” Her statement also suggested that others’ perception would not deter her from pursuing her objectives. For Anna, concern about her coach’s negative evaluation served as a motivator to invest more effort upon returning to her sport. When asked whether these concerns could positively influence her recovery or return to sport, Anna responded: “I think so, because the fact that she may have seen some of the less good things about me, it means that I’ll put more effort into making my practice better, which will allow me to then play better, to change the impression she might have had of me.” Her comments reflected a determination to alter her coach’s potentially limited or unfavorable perception—one shaped by seeing her only during her injury period, rather than at her full potential or capability.

### 3.4. Self-Presentation Concerns’ Management Strategies

This theme addresses coping strategies and techniques used by injured adolescent athletes when experiencing self-presentation concerns. Athletes reported employing various coping strategies to manage their self-presentation concerns, ranging from seeking social support from relevant others, trusting the expertise of healthcare professionals and coaches, or, in some cases, having no specific coping strategies. Several athletes reported that seeking social support from individuals they considered important helped to manage their self-presentation concerns. As indicated previously, individuals perceived as “important” varied from one athlete to another, depending on the personal value and trust placed in those relationships. Being able to talk openly about their experiences and the psychological impact of self-presentation concerns was particularly beneficial for many. For example, when concerned about the fact that her teammates believed she was using her injury to avoid hard training, Olivia shared: “I talked to my coach about it. She threw a word in front of everyone. She said: ‘It’s not cool what’s happening to Olivia. I don’t think anyone would like to be in this situation right now.’ Then after that, [the situation] improved.” Similarly, when concerned that her friends might see her differently since her injury or believe she was using her injury as an excuse to avoid training, Emily emphasized the importance of honest communication: “I tell my friends about it, and I can’t wait to come back. I really say what I think. Thanks to that, I think they haven’t changed their point of view [of me].”

For other athletes, simply discussing their concerns with relevant others they knew that they could confide in was helpful. For example, Marie mentioned: “I talked about it with my physical trainer, who is also a person I could confide in”. She also added: “I also try to talk to my sister about it especially. I would say more to my sister, she is the person I talked about it with the most. It helped a lot to talk to her too.” Valerie shared the emotional relief she felt from speaking with her parents, as she was no longer able to rely on her usual coping strategies:

I like talking to my parents about it, it helps me because it lets me vent a little. What’s also hard is that before, when I was going through something, I would just go outside and play basketball or soccer, but now I can’t do that anymore. I like to talk about it. It makes me feel better.

Additionally, trust in the expertise of healthcare professionals and coaches–particularly regarding recovery and regaining pre-injury performance levels–played an important role for some participants. Liam described relying on professionals’ guidance when facing negative evaluation from teammates about his performance when returning to sport. He stated:

I try to tell myself, it’s the professionals who told me that I was going to be okay, so I tell myself that I’m going to trust those who really know. My teammates, of course I’m interested in what they think, but in such specific cases, I tell myself that they don’t necessarily know everything, and then it’s my professional who knows. I don’t feel stressed.

His response suggests that the knowledge and validation of healthcare professionals and coaches helped diminish the athlete’s stress associated with teammates’ negative evaluations about their ability to regain pre-injury capabilities.

Finally, some athletes reported having no specific coping strategies for dealing with self-presentation concerns. When asked about the strategies they used when experiencing self-presentation concerns, one athlete described a passive approach, indicating she simply endured the discomfort rather than actively addressing it. For instance, Sarah stated: “I didn’t really have a strategy, I think… I rather let it go…” Similarly, Margot commented: “I don’t know if I really have a strategy […] I think I do it more by feeling and yes… No, I think I don’t necessarily have a strategy, and I don’t necessarily manage it much.” Although some athletes did mention efforts to distract themselves as a strategy, these efforts were often perceived as ineffective or difficult to maintain. Eve illustrated this well: “I’m trying to distract myself, but it’s really hard. I always look at it in the back of my mind”. Elodie described using distraction by focusing on activities unrelated to her sport, such as playing non-sport games, to take her mind off her concerns: “I try to think about something else, like playing [other non-sport activities].” Benjamin appeared uncertain or contradictory in his response: “I don’t have too many. I’m just trying to… […] I don’t do too many things, just everything. […] I try to think less about what they think of me. I try as much as possible to just be in my zone and ignore what they’re thinking”.

## 4. Discussion

To our knowledge, this is the first study addressing self-presentation concerns in injured adolescent athletes using a qualitative approach. Interviews with 14 injured adolescent athletes led to identifying four themes related to how self-presentation concerns influenced their injury experiences: (1) the nature of self-presentation concerns, (2) the factors influencing self-presentation concerns, (3) the implications of self-presentation concerns for athletes’ rehabilitation and return to sport, and finally (4) self-presentational management strategies. This study enhances understanding of the complexities of the concerns faced by injured adolescent athletes during rehabilitation and return to sport, providing a valuable foundation for adapting or developing new interventions to manage these concerns.

In terms of the nature of self-presentation concerns (theme 1), findings from this study revealed new insights suggesting that the presence of self-presentation concerns extend beyond the competitive setting, to adolescent athletes’ injury rehabilitation and their return to sport [40]. A novel concern raised by participants pertained to worries about being perceived as “faking” one’s injury or using it as an excuse to avoid training or competing. This concern seemed to arise when athletes felt that relevant others doubted the presence of their injury or when they were unable to clearly communicate their condition to dispel such doubts. These findings align with Roderick [53], who reported that professional footballers feared that others would see them as feigning their injury to avoid training or playing. In the current study and in Roderick’s, athletes were concerned about being seen as “faking” injury, despite being legitimately injured and expressing to others an intent to return to sport. These findings are in contrast to those of Silver [54] and Caron et al. [55], in which some young athletes engaged in malingering by feigning a concussion as a way to avoid returning to sports they no longer wanted to participate in, despite receiving medical clearance to do so. In the present investigation, it was unclear whether relevant others doubted the veracity of athletes’ injury status claims or whether such preoccupations existed only in the minds of participants, given that only athlete perspectives were ascertained. Future research could benefit from capturing the perspectives of relevant others to gain a clearer understanding of whether and under what circumstances an athlete’s claims about their injury status are likely to be deemed credible or dismissed. Previous research suggests that athlete indications about injury may be more likely to be considered “legitimate” when coaches or physical training staff members have a history with athletes and consider them “dedicated” to their sport [56,57,58]. Given the cultural ethos in many sports in which athletes are lauded for and encouraged to push their limits, to disregard pain and injury, and to expedite their resumption of sporting activities [59,60,61,62], it may be important to consider such sporting norms and sub-cultural values when investigating coach–athlete interactions, communications and acceptance or denial of athletes’ injury assertions. Ultimately, findings from this investigation suggest that athletes may form beliefs about the extent to which others believe their injury reports. Healthcare providers, coaches and others attempting to promote athlete welfare and to alleviate athlete worries should therefore be aware of this potentially prominent concern rooted in self-presentational interests. 

Another key finding from the present study in relation to theme 1, related to participants’ concerns about others’ perception of their athletic competence, particularly in regard to a return to sport following rehabilitation. Participants worried about being perceived as weak, less competent, or underperforming. Such concerns are consistent with previous research documenting injured athletes’ anxieties about appearing unfit, unskilled, or diminished in ability after an extended absence from sport [38,40,41,63]—perhaps, a reflection of the pressure to re-establish one’s reputation. Participants in the current investigation also reported experiencing anxiety about disappointing others once they resumed their sport participation. These concerns align with previous research highlighting self-presentation concerns among athletes during the return to competition phase [38]. Podlog and Eklund [38] found that high-level athletes were concerned about failing to meet expectations, letting down teammates or coaches, and protecting their reputations. While some participants in the present study were also primarily concerned about disappointing coaches or sport staff, it was evident that other athletes were particularly worried about letting down parents. The latter finding is interesting in light of developmental research indicating that adolescents may become increasingly concerned with external sources of validation from peers and adults [64,65]. Conversely, our findings regarding adolescent preoccupations with disappointing their parents are consistent with sociological research, in which young persons often expressed worries about continuing and maintaining their athletic careers and performance due to the significant investment made by their parents [66,67]. The extent to which athletes are concerned about disappointing coaches versus parents may, to some extent, be a function of the closeness of those respective relationships. For instance, worries about letting down parents may be diminished in instances where athletes experience close relationships with their coaches. Alternatively, close parent–child relations may help attenuate athlete worries about letting coaches down. Further research is needed to corroborate findings regarding the target of athletes’ self-presentational worries and to examine whether strong coach–athlete relationships help offset dyadic concerns about parents or vice versa.

Regarding theme 2, a novel factor influencing the extent to which adolescents appeared to experience self-presentation concerns pertained to the degree of closeness with significant others. In some cases, close relationships intensified athletes’ concerns, while in others, a lack of closeness heightened uncertainty and worry. Research outside the sport injury context has highlighted the importance of the coach–athlete relationship for a range of well-being [68,69], performance [70], and participation outcomes [71,72]. Findings from the current study extend these findings into the sport injury realm and suggest that coach–athlete relationships may have a significant bearing on the extent to which injured adolescents experience self-presentational worries. Specifically, perceived negative evaluations by coaches during injury contributed to fears about future involvement in sport, especially concerning one’s playing time and the athlete’s role on the team. Participants indicated that being injured could negatively alter how coaches viewed them, potentially limiting future opportunities. This concern was particularly pronounced among athletes returning to teams with new coaches. In such cases, the absence of prior rapport made it difficult for athletes to manage perceptions. Findings suggest that providing structured opportunities for communication between returning athletes and coaches, especially when the coach is new, may help mitigate these concerns and support reintegration into the team environment. Additional research examining this contention is warranted.

In relation to theme 3, the current study also uncovered important findings regarding the implications of self-presentation concerns. A variety of negative emotions, such as anger, fear, sadness, and anxiety were all reported. These findings support the notion that self-presentation concerns can adversely impact athletes’ affective states, with anxiety surfacing when athletes are negatively perceived by significant others and lack the ability to make desired impressions [35,73,74]. While participants openly discussed these emotional reactions, they provided limited insight into how these feelings influenced their rehabilitation or return to sport. Further exploration is needed to better understand the potential behavioral impact of these emotions on athletes’ rehabilitation and return to sport outcomes.

This investigation further revealed novel benefits of self-presentation concerns (theme 3), in particular, as a source of motivation to disprove others’ doubts about one’s capacity to effectively recover. That is, self-presentation concerns appeared to act as a constructive motivational force initiating participants’ proactive rehabilitation behaviors. Participants expressed that negative evaluations from others spurred their motivation to engage in recovery efforts to be viewed as competent in the eyes of others. This finding is in line with Leary’s [35] proposition that self-presentation concerns may account for athletes’ drive to work hard during rehabilitation in order to preserve or demonstrate a valued social identity. It also aligns with sociological research suggesting that injuries can act as a catalyst for deeper engagement in sport, potentially reinforcing or sustaining athletic commitment [67]. Additionally, this result is consistent with the work of Jones and Pittman [75] who suggested that individuals may be motivated to appear competent and to be respected. While previous research with injured elite adolescent athletes noted increased motivation during the injury period [34], this motivation was not necessarily driven by self-presentation concerns or the wish to prove others wrong, as observed in the present study. The findings from this study also diverge from youth development literature that typically associates adolescents’ self-presentational worries–such as the desire to avoid embarrassment or negative peer evaluation–with engagement in health-jeopardizing behaviors [76,77]. This study presents a more positive implication of self-presentation concerns, differing from previous work by Podlog, Gao, Kenow, Kleinert, Granquist, Newton and Hannon [41], who found that such concerns–particularly fears of appearing athletically untalented, physically unfit, or lacking energy–were linked to unhealthy behaviors, such as ignoring medical advice and risking a premature return to sport. Findings from the current study, therefore, indicate the possibility that self-presentational concerns may be leveraged to convert athlete worries into sources of productive action.

Finally, participants suggested different strategies for managing self-presentation concerns (theme 4), including seeking support from significant others, placing trust in the expertise of healthcare professionals and coaches, and in some instances, not employing any specific coping methods. These findings align with previous research emphasizing the critical role of interpersonal relationships in the injury recovery process [78,79,80]. Support from coaches, physiotherapists, and peers can reduce emotional distress, enhance confidence in the rehabilitation process (e.g., knowledge of recovery timeline, exercises), and increase adherence to prescribed rehabilitation regimens. These relationships can also help maintain a sense of identity and belonging, which is often disrupted during injury. Consistent with previous findings, the absence of coping strategies among adolescent athletes may be a reflection of their developmental stage, where younger individuals often have limited experience navigating significant stressful events such as severe injuries [81,82,83]. The ability to effectively manage self-presentation concerns appeared to vary among individuals, highlighting the need for more targeted support and guidance during the recovery process.

### Limitations and Future Directions

While this investigation revealed further insights into the nature, precursors, and implications of injured adolescent self-presentation concerns, this study is not without limitations. One limitation was the absence of pilot interviews with participants, which could have helped refine or generate more insightful questions. Another limitation relates to the depth of participants’ responses during interviews. The lack of depth may have in part been related to the age of participants, some of whom provided brief or limited responses and were not particularly verbal or expressive. Additionally, several participants needed more time to reflect on the questions, which often required introspection. As a result, certain interviews were relatively short and lacked rich, detailed narratives. Researchers are therefore encouraged to employ novel methodologies, such as vignettes or video diaries, to further explore injured adolescents’ self-presentation concerns. Vignettes may provide space for reflection and may encourage participants to relate given scenarios to their own experiences over time [84]. Video diaries, including prompts or directed questions, may also offer a valuable complementary approach [85]. In particular, video diaries may help to capture the immediacy of participants’ experiences, as they can record their thoughts, feelings, and behaviors shortly after they occur.

Another limitation of the current work pertained to the variable timing of participants’ injury. Although all participants met the inclusion criteria of being currently injured, some had been injured or in rehabilitation for longer periods than others (e.g., several months versus a few weeks). These differing time lapses may have made it harder for some participants to recall their experiences, thoughts, feelings, and behaviors during the injury period with clarity. Many participants mentioned that their concerns were more prominent earlier in the injury and recovery process. As this was an exploratory study, the presence of self-presentation concerns was identified, but varied across participants and should be further explored. Finally, our sample included an imbalance in the gender of participants (2 males and 12 females). A predominantly female sample may be considered a strength of the present study, given that past research often includes higher proportions of male athletes [86]. That said, it may be the case that our findings reflect concerns that may be more evident among injured female athletes. Research outside the sport injury context suggests that females may experience self-presentation concerns to a greater extent than males [87,88,89]. Studies indicate that women tend to place a higher priority on creating a positive self-image compared to men [87] and exhibit stronger self-presentational motives in everyday interactions, being more conscious of how they are perceived in various social contexts [89]. Additionally, women are often socialized to emphasize appearance and seek social approval, which may lead them to engage more frequently in self-presentation behaviors [88]. However, these gender differences require further empirical scrutiny within sports injury settings.

In addition to the aforementioned suggestions for future research, a number of fruitful avenues for further scholarship remain. Questions such as, “To what extent does gender, sport type (team or individual), or personality factors influence injured adolescents’ self-presentation concerns”? “How can injured adolescent athletes be better equipped to self-manage their self-presentation concerns”? or “How can injured adolescent athletes be better supported by relevant others in managing self-presentation concerns”? Further scholarship is also needed to determine the efficacy of age-appropriate interventions such as cognitive restructuring, goal-setting techniques and the provision of social support for minimizing the detrimental impacts of self-presentation concerns. For instance, cognitive restructuring techniques enabling adolescents to become more aware of their concerns, either independently or with the help of relevant others, such as sports medicine professionals, may assist injured athletes in reframing their perspectives and concerns by redirecting their attention to intrinsic motivations (e.g., excitement of participation, social advantages of sports involvement). Additionally, goal setting focused on self-referent, short-term objectives could be useful in diminishing self-presentation concerns and facilitating athletes’ confidence in regaining pre-injury capabilities [90,91]. Furthermore, social support may be a valuable strategy that helps athletes navigate their injury recovery while being surrounded by helpful relevant others. These strategies could all be tested in future research.

## 5. Conclusions

This study explored the injury experiences of adolescent athletes in regard to self-presentation concerns. Findings shed a more nuanced light on the types of self-presentational worries occupying the minds of young injured athletes, the importance of coach–athlete relationships in mitigating or increasing such concerns and key implications of such concerns (e.g., negative emotions and enhanced motivation to prove others wrong). Although the aim of our qualitative investigation was not to generalize our findings to other athlete populations, we believe that our findings may resonate with other injured adolescent populations or sports other than those investigated in the current study. As such, results from this investigation can be used to help inform age-appropriate interventions aimed at reducing the deleterious impact of self-presentation concerns and optimizing adolescent athletes’ rehabilitation and return to sport. Further research is needed to explore additional influencing factors and potential implications of self-presentation concerns as well as the efficacy of behavioral interventions.

## Figures and Tables

**Table 1:** Participants’ Demographics.

Pseudonym	Sex	Age	Sport	Level of Competition	Injury	Weeks away from Sport	First Serious Injury
Liam	M	15	Ice Hockey	National	Labrum tear/Humeral glans fracture	4	Yes
Emily	F	16	Judo	International	Elbow sprain	5	Yes
Margot	F	16	Cycling	National	Calves pain after labrum surgery	52	No
Sarah	F	14	Acrobatic Skiing	National	ACL rupture	39	Yes
Charlotte	F	17	Basketball	Regional	ACL rupture	52	Yes
Olivia	F	14	Softball	Regional	Labrum tear	17	Yes
Emma	F	16	Judo	National	ACL sprain	9	No
Eve	F	16	Soccer	National	ACL rupture	7	Yes
Benjamin	M	14	Soccer	Regional	Meniscus rupture	29	Yes
Elodie	F	11 *	Gymnastics	International	Wrists stress fracture	4	No
Marie	F	18 **	Soccer	National	ACL rupture/Meniscus rupture	67	Yes
Anna	F	15	Soccer	National	External ligaments sprain	39	No
Evelyn	F	16	Diving	International	Patella osteochondritis dissecans	19	No
Valerie	F	14	Basketball	Regional	Severe meniscus irritation	15	No

* At 11 years old, Elodie was slightly below our minimum age criteria of 12 years; however, due to initial recruitment challenges, we nonetheless decided to include Elodie’s data in the final analysis of transcripts. ** At 18 years old, Marie was slightly above our maximum age criteria of 17 years; however, due to initial recruitment challenges, we nonetheless decided to include Marie’s data in the final analysis of transcripts.

## Data Availability

The raw data supporting the conclusions of this article will be made available by the authors on request.

## References

[1] Kamphoff C.S., Thomae J., Hamson-Utley J.J. (2013). Integrating the Psychological and Physiological Aspects of Sport Injury Rehabilitation. Rehabilitation Profiling and Phases of Rehabilitation.

[2] Bauman J. (2005). Returning to Play: The Mind Does Matter. Clin. J. Sport Med..

[3] Charlesworth H., Young K. (2004). Why English female university athletes play with pain: Motivations and rationalizations. Sporting Bodies, Damaged Selves: Sociological Studies of Sports-Related Injury.

[4] Norlin T., Tranaeus F.U., Alricsson M. (2016). Barriers to and possibilities of returning to play after a severe soccer injury: A qualitative study. Eur. J. Physiother..

[5] Corbillon F., Crossman J., Jamieson J. (2008). Injured athletes’ perceptions of the social support provided by their coaches and teammates during rehabilitation. J. Sport Behav..

[6] Brewer B.W. (2007). Psychology of sport injury rehabilitation. Handbook of Sport Psychology.

[7] Johnston L.H., Carroll D. (1998). The Provision of Social Support to Injured Athletes: A Qualitative Analysis. J. Sport Rehabil..

[8] Udry E. (1997). Coping and Social Support among Injured Athletes Following Surgery. J. Sport Exerc. Psychol..

[9] Brewer B.W. (2010). The role of psychological factors in sport injury rehabilitation outcomes. Int. Rev. Sport Exerc. Psychol..

[10] Granquist M.D., Brewer B.W. (2013). Psychological aspects of rehabilitation adherence. The Psychology of Sport Injury and Rehabilitation.

[11] Leddy M.H., Lambert M.J., Ogles B.M. (1994). Psychological Consequences of Athletic Injury among High-Level Competitors. Res. Q. Exerc. Sport.

[12] Forsdyke D., Smith A., Jones M., Gledhill A. (2016). Psychosocial factors associated with outcomes of sports injury rehabilitation in competitive athletes: A mixed studies systematic review. Br. J. Sports Med..

[13] Tripp D.A., Stanish W., Ebel-Lam A., Brewer B.W., Birchard J. (2007). Fear of reinjury, negative affect, and catastrophizing predicting return to sport in recreational athletes with anterior cruciate ligament injuries at 1 year postsurgery. Rehabil. Psychol..

[14] Kvist J., Ek A., Sporrstedt K., Good L. (2005). Fear of re-injury: A hindrance for returning to sports after anterior cruciate ligament reconstruction. Knee Surg. Sports Traumatol. Arthrosc..

[15] Mankad A., Gordon S., Wallman K. (2009). Perceptions of Emotional Climate Among Injured Athletes. J. Clin. Sport Psychol..

[16] Tracey J. (2003). The Emotional Response to the Injury and Rehabilitation Process. J. Appl. Sport Psychol..

[17] Walker N., Thatcher J., Lavallee D. (2010). A preliminary development of the Re-Injury Anxiety Inventory (RIAI). Phys. Ther. Sport.

[18] Weiss M.R. (2003). Psychological Aspects of Sport-Injury Rehabilitation: A Developmental Perspective. J. Athl. Train..

[19] Weiss M.R., Raedeke T.D. (2004). Developmental Sport and Exercise Psychology: Research Status on Youth and Directions Toward a Lifespan Perspective.

[20] Steinberg L., Morris A.S. (2001). Adolescent Development. Annu. Rev. Psychol..

[21] Spear L.P. (2000). The adolescent brain and age-related behavioral manifestations. Neurosci. Biobehav. Rev..

[22] Steinberg L., Monahan K.C. (2007). Age differences in resistance to peer influence. Dev. Psychol..

[23] Haraldsdottir K., Watson A.M. (2021). Psychosocial Impacts of Sports-related Injuries in Adolescent Athletes. Curr. Sports Med. Rep..

[24] Friedman H.L. (1989). The health of adolescents: Beliefs and behaviour. Soc. Sci. Med..

[25] Martin K.A., Leary M.R. (2001). Self-presentational determinants of health risk behavior among college freshmen. Psychol. Health.

[26] Brewer B.W. (2003). Developmental Differences in Psychological Aspects of Sport-Injury Rehabilitation. J. Athl. Train..

[27] Brewer B.W., Linder D.E., Phelps C.M. (1995). Situational Correlates of Emotional Adjustment to Athletic Injury. Clin. J. Sport Med..

[28] Brewer B.W., Cornelius A.E., Van Raalte J.L., Petitpas A.J., Sklar J.H., Pohlman M.H., Krushell R.J., Ditmar T.D. (2003). Age-Related Differences in Predictors of Adherence to Rehabilitation After Anterior Cruciate Ligament Reconstruction. J. Athl. Train..

[29] Tripp D.A., Stanish W.D., Reardon G., Coady C., Sullivan M.J. (2003). Comparing Postoperative Pain Experiences of the Adolescent and Adult Athlete After Anterior Cruciate Ligament Surgery. J. Athl. Train..

[30] Udry E., Donald Shelbourne K., Gray T. (2003). Psychological Readiness for Anterior Cruciate Ligament Surgery: Describing and Comparing the Adolescent and Adult Experiences. J. Athl. Train..

[31] Neal T.L., Diamond A.B., Goldman S., Liedtka K.D., Mathis K., Morse E.D., Putukian M., Quandt E., Ritter S.J., Sullivan J.P. (2015). Interassociation Recommendations for Developing a Plan to Recognize and Refer Student-Athletes With Psychological Concerns at the Secondary School Level: A Consensus Statement. J. Athl. Train..

[32] Park A.L., Furie K., Wong S.E. (2023). Stronger Athlete Identity Is a Risk Factor for More Severe Depressive Symptoms After Musculoskeletal Injury in Pediatric Athletes: A Systematic Review. Curr. Rev. Musculoskelet. Med..

[33] Padaki A.S., Noticewala M.S., Levine W.N., Ahmad C.S., Popkin M.K., Popkin C.A. (2018). Prevalence of Posttraumatic Stress Disorder Symptoms Among Young Athletes After Anterior Cruciate Ligament Rupture. Orthop. J. Sports Med..

[34] Von Rosen P., Anders K., Cecilia F., Anna F., Heijne A. (2018). Young, talented and injured: Injury perceptions, experiences and consequences in adolescent elite athletes. Eur. J. Sport Sci..

[35] Leary M.R. (1992). Self-Presentational Processes in Exercise and Sport. J. Sport Exerc. Psychol..

[36] Schlenker B.R. (1980). Impression Management.

[37] Podlog L., Dimmock J., Miller J. (2011). A review of return to sport concerns following injury rehabilitation: Practitioner strategies for enhancing recovery outcomes. Phys. Ther. Sport.

[38] Podlog L., Eklund R.C. (2006). A Longitudinal Investigation of Competitive Athletes’ Return to Sport Following Serious Injury. J. Appl. Sport Psychol..

[39] Bailey K.A., Gammage K.L., van Ingen C., Ditor D.S. (2016). Managing the stigma: Exploring body image experiences and self-presentation among people with spinal cord injury. Health Psychol. Open.

[40] Driediger M.V., McKay C.D., Hall C.R., Echlin P.S. (2017). A qualitative examination of women’s self-presentation and social physique anxiety during injury rehabilitation. Physiotherapy.

[41] Podlog L., Gao Z., Kenow L., Kleinert J., Granquist M., Newton M., Hannon J. (2013). Injury Rehabilitation Overadherence: Preliminary Scale Validation and Relationships With Athletic Identity and Self-Presentation Concerns. J. Athl. Train..

[42] Sparkes A.C., Smith B. (2013). Qualitative Research Methods in Sport, Exercise and Health: From Process to Product.

[43] Creswell J.W. (2013). Qualitative Inquiry & Research Design: Choosing Among Five Approaches.

[44] Tamminen K.A., Poucher Z.A. (2020). Research philosophies. The Routledge International Encyclopedia of Sport and Exercise Psychology.

[45] Bahr R., Clarsen B., Derman W., Dvorak J., Emery C.A., Finch C.F., Hägglund M., Junge A., Kemp S., Khan K.M. (2020). International Olympic Committee Consensus Statement: Methods for Recording and Reporting of Epidemiological Data on Injury and Illness in Sports 2020 (Including the STROBE Extension for Sports Injury and Illness Surveillance (STROBE-SIIS)). Orthop. J. Sports Med..

[46] Bianco T., Malo S., Orlick T. (1999). Sport Injury and Illness: Elite Skiers Describe Their Experiences. Res. Q. Exerc. Sport.

[47] Ahmed S.K. (2025). Sample size for saturation in qualitative research: Debates, definitions, and strategies. J. Med. Surg. Public Health.

[48] Fletcher D., Arnold R. (2011). A Qualitative Study of Performance Leadership and Management in Elite Sport. J. Appl. Sport Psychol..

[49] Dimmock J.A., Howle T.C., Jackson B. (2020). Self-Presentation in Sport and Exercise. Handbook of Sport Psychology.

[50] Braun V., Clarke V. (2006). Using thematic analysis in psychology. Qual. Res. Psychol..

[51] Braun V., Clarke V., Weate P. (2016). Using thematic analysis in sport and exercise research. Routledge Handbook of Qualitative Research in Sport and Exercise.

[52] Smith B., McGannon K.R. (2018). Developing rigor in qualitative research: Problems and opportunities within sport and exercise psychology. Int. Rev. Sport Exerc. Psychol..

[53] Roderick M. (2006). The Work of Professional Football: A Labour of Love?.

[54] Silver J.M. (2012). Effort, exaggeration and malingering after concussion. J. Neurol. Neurosurg. Psychiatry.

[55] Caron J.G., Cadotte G., Collict C., Josee van Ierssel J., Podlog L. (2023). Exploring the Factors Involved in Being “Ready” to Return to Sport Following a Concussion. Clin. J. Sport Med..

[56] van Wilgen C.P., Verhagen E.A. (2012). A qualitative study on overuse injuries: The beliefs of athletes and coaches. J. Sci. Med. Sport.

[57] Kristensen O.S. (2004). Changing goals and intentions among participants in a neuropsychological rehabilitation programme: An explorative case study evaluation. Brain Inj..

[58] Rees H., Matthews J., McCarthy Persson U., Delahunt E., Boreham C., Blake C. (2022). The knowledge and attitudes of field hockey athletes to injury, injury reporting and injury prevention: A qualitative study. J. Sci. Med. Sport.

[59] Curry T.J. (1993). A Little Pain Never Hurt Anyone: Athletic Career Socialization and the Normalization of Sports Injury. Symb. Interact..

[60] Jessiman-Perreault G., Godley J. (2016). Playing through the pain: A university-based study of sports injury. Adv. Phys. Educ..

[61] Kroshus E., Garnett B.R., Baugh C.M., Calzo J.P. (2015). Social norms theory and concussion education. Health Educ. Res..

[62] Wiese-Bjornstal D.M. (2018). Sociocultural Aspects of Sport Injury and Recovery. Oxf. Res. Encycl. Psychol..

[63] McGowan E., Prapavessis H., Wesch N. (2008). Self-Presentational Concerns and Competitive Anxiety. J. Sport Exerc. Psychol..

[64] Birkeland M.S., Breivik K., Wold B. (2014). Peer Acceptance Protects Global Self-esteem from Negative Effects of Low Closeness to Parents During Adolescence and Early Adulthood. J. Youth Adolesc..

[65] Guyer A.E., Caouette J.D., Lee C.C., Ruiz S.K. (2014). Will they like me? Adolescents’ emotional responses to peer evaluation. Int. J. Behav. Dev..

[66] Lefèvre N. (2010). Construction sociale du don et de la vocation de cycliste. Sociétés Contemp..

[67] Forté L. (2020). Sport as a vocation: The effects of injury on the socialization processes involved in the production of sporting elites. Int. Rev. Sociol. Sport.

[68] Coussens A.H., Stone M.J., Donachie T.C. (2025). Coach-athlete relationships, self-confidence, and psychological wellbeing: The role of perceived and received coach support. Eur. J. Sport Sci..

[69] Davis L., Jowett S. (2014). Coach–athlete attachment and the quality of the coach–athlete relationship: Implications for athlete’s well-being. J. Sports Sci..

[70] Jowett S., Cockerill I.M. (2003). Olympic medallists’ perspective of the althlete–coach relationship. Psychol. Sport Exerc..

[71] Fraser-Thomas J., Côté J., Deakin J. (2008). Understanding dropout and prolonged engagement in adolescent competitive sport. Psychol. Sport Exerc..

[72] Wekesser M.M., Harris B.S., Langdon J., Wilson C.H. (2021). Coaches’ impact on youth athletes’ intentions to continue sport participation: The mediational influence of the coach–athlete relationship. Int. J. Sports Sci. Coach..

[73] Leary M.R., Kowalski R.M. (1995). The self-presentation model of social phobia. Social Phobia: Diagnosis, Assessment, and Treatment.

[74] Wilson P., Eklund R.C. (1998). The Relationship between Competitive Anxiety and Self-Presentational Concerns. J. Sport Exerc. Psychol..

[75] Jones E., Pittman T. (1982). Toward a general theory of strategic self-presentation. Psychol. Perspect. Self.

[76] Shroff H., Thompson J.K. (2006). Peer influences, body-image dissatisfaction, eating dysfunction and self-esteem in adolescent girls. J. Health Psychol..

[77] Siraj R., Najam B., Ghazal S. (2021). Sensation Seeking, Peer Influence, and Risk-Taking Behavior in Adolescents. Educ. Res. Int..

[78] Bianco T., Eklund R.C. (2001). Conceptual Considerations for Social Support Research in Sport and Exercise Settings: The Case of Sport Injury. J. Sport Exerc. Psychol..

[79] Clement D., Shannon V.R. (2011). Injured athletes’ perceptions about social support. J. Sport Rehabil..

[80] Yang J., Schaefer J.T., Zhang N., Covassin T., Ding K., Heiden E. (2014). Social support from the athletic trainer and symptoms of depression and anxiety at return to play. J. Athl. Train..

[81] Nicholls A., Polman R., Morley D., Taylor N.J. (2009). Coping and coping effectiveness in relation to a competitive sport event: Pubertal status, chronological age, and gender among adolescent athletes. J. Sport Exerc. Psychol..

[82] Tamminen K.A., Holt N.L. (2010). A meta-study of qualitative research examining stressor appraisals and coping among adolescents in sport. J. Sports Sci..

[83] Manuel J.C., Shilt J.S., Curl W.W., Smith J.A., Durant R.H., Lester L., Sinal S.H. (2002). Coping with sports injuries: An examination of the adolescent athlete. J. Adolesc. Health.

[84] Barter C., Renold E. (2000). ‘I wanna tell you a story’: Exploring the application of vignettes in qualitative research with children and young people. Int. J. Soc. Res. Methodol..

[85] Flanagan S.M., Greenfield S., Coad J., Neilson S. (2015). An exploration of the data collection methods utilised with children, teenagers and young people (CTYPs). BMC Res. Notes.

[86] Laxdal A. (2023). The sex gap in sports and exercise medicine research: Who does research on females?. Scientometrics.

[87] Haferkamp N., Eimler S.C., Papadakis A.-M., Kruck J.V. (2012). Men Are from Mars, Women Are from Venus? Examining Gender Differences in Self-Presentation on Social Networking Sites. Cyberpsychol. Behav. Soc. Netw..

[88] Nor N.F.M., Iqbal N., Shaari A.H. (2025). The Role of False Self-Presentation and Social Comparison in Excessive Social Media Use. Behav. Sci..

[89] Leary M.R., Nezlek J.B., Downs D., Radford-Davenport J., Martin J., McMullen A. (1994). Self-presentation in everyday interactions: Effects of target familiarity and gender composition. J. Pers. Soc. Psychol..

[90] Brewer B.W., Redmond C.J., Brewer B.W., Redmond C.J. (2017). Psychology of Sport Injury. Psychology of Sport Injury.

[91] Nippert A.H., Smith A.M. (2008). Psychologic Stress Related to Injury and Impact on Sport Performance. Phys. Med. Rehabil. Clin. N. Am..

